# Tetanus

**Published:** 1891-09

**Authors:** Claude D. Morris


### TETANUS.*

By Dr. Claude D. Morris.

Tetanus is a constitutional traumatic infective disease which acts upon the central nervous system primarily, and which is clinically characterized by spasm and rigidity of definite muscular groups, as a physiological result of the ptomaines of bacillus tetani.

Bacteriological studies and the classification of this disease as of an infectious character is of recent date. In 1859 Betali related the case of a bull that died of tetanus after castration, several slaves ate of the flesh of the dead animal, and of these three were seized in a few days with tetanus, and two of them died. In 1870 Angar reported a case in which a horse had spontaneous tetanus, after which three puppies which had been in the same stable were also affected. Larger, in 1853, saw a woman who had a fall while cleaning a farm-yard, causing a slight wound of the elbow, four weeks later she was seized with tetanus, and on investigation it was found that a horse affected with that disease had been in a stable opening into the yard, where she fell. He also mentioned another circumstance, in a small village, where tetanus was previously unknown, five cases appeared in eighteen months under quite different climatic conditions. Of these, one had been taken to a hospital, after which two others in the same ward became affected with the disease.

The oxogenious origin of the disease has been proven by Nicolaier, who produced the disease by injecting into the tissue of the animals bacilli taken from earth.

Rosenbach found the same bacillus in the pus of a patient suffering from traumatic tetanus.

The identity of the bacillus of tetanus, with Nicolaier's bacillus of earth tetanus, was demonstrated by Koch in 1887.

The physical characters of bacillus tetanus, stained with fuchsin 1–1000, are those of an anærobic micro-organism, which presents more than ordinary life, with a spore at the extremity having the appearance of a broken drum-stick. Kitasato, in speaking of the bacillus, as to its function of reproduction, says, that the bacillus tetani produces spores in thirty hours, in culture kept at a temperature of the body, they possess great resistance to heat, as

* Read before the New York State Veterinary Medical Society.

they have been found active after an exposure of an hour to 80°C. of moist heat, but they are destroyed by placing them in a sterilizer heated to 100°C. for five minutes.

The bacillus has been found in different kinds of surface soil and in street dust. By man it has been found in tetanic patients in the wound-secretions, in the nerves leading from the seat of infections and in the spinal cord.

### Cultivations, Inoculations, Experiments, Etc.

It has been a question of dispute among pathologists as to the specific cause of the disease. The same questions have been raised in connection with the pathogenic action of the bacillus tetanus, as with pus-microbes, *i. e.* Is the disease of which it was the specific cause due to the presence of the microbes, or the ptomaines which it elaborates in the tissues? It has therefore been demonstrated beyond a doubt that the ptomaines of the bacillus of tetanus cause tetanic convulsions.

However, symptoms in many respects analagous to that of tetanus, can be produced with strychina, when given in toxic doses. If this and other drugs belonging to the same group can act upon the spinal cord in such a manner as to cause spasms and muscular rigidity, we should therefore expect that if the microbes of tetanus produced ptomaines in the tissues, these might produce the same effect on the cords, and that the symptoms are produced by them and not by the direct actions of the microbes.

It is conceded by nearly all authorities that there are but few bacilli present in the blood of tetanic patients, and in many instances in which the disease was produced artificially, the blood was often found sterile. On the other hand, more microbes have been found at the seat of primary infections and in the tissues, between it and the spinal cord, than in the blood itself.

Perhaps stronger proof than any as yet brought forward. to show that the direct cause of the disease is the product of the microbes, and not the microbes themselves, is the experiments made by Brieger, who has succeeded in isolating four toxic substances from mixed cultures of the tetanus bacillus in sterilized emulsion of meat.

First, Tetanus, when administered subcutaneously in mice produced the characteristic symptoms of tetanus.

Second, Tetanotoxin causes first, tremors, followed by convulsions and paralysis.

Third, The muriate of toxin produces well-marked symptoms of tetanus, besides exciting the lachrymal and salivary glands to increased functual activity, the last spasmotoxin, also produces clonic and toxic spasms which prostate the animal at once.

As to the etiology of tetanus, it has been clearly demonstrated beyond all doubt, that the disease is due to microbe influences, whether ushered in as a traumatic or idiopathic disease, or artificially produced by infections of wound secretions of tetanic patients, or by using mixed or pure cultures. The essential cause of the disease is the bacillus first discovered by Nicolaier in earth, and by Rasenbach in the wound secretions of a tetanic patient. The period of incubation seems to be extremely variable in both man and animals. In some cases existing only twenty-four hours, in others lasting weeks, between the time of infection and the first manifestations of the disease.

This may be accounted for, first, the number of bacilli introduced into the system may be so small that a longer time is necessary before the disease is manifest. Second, the character and location of a surgical operation in many instances acts as an infectious atrium. Also the anatomical characteristics of the tissues surrounding it may influence the time which is necessary to develop the disease, and further, the investigation of Brieger's, have shown that the tetanic convulsions are produced by infections of tetanus and of the toxic ptomaines, derived from cultures of the bacillus of tetanus. It is more than probable that the active symptoms of tetanus are due, not to the presence in the tissues of the bacillus, but to the toxic action of the ptomaines on the spinal-cord, so that the duration of the period of incubation is further modified by the capacity of the infected tissues to yield to different ptomaines, as in the second instance, the character of certain surgical operations play an important part as an infectious atrium in the practice of veterinary surgery.

My experience leads me to believe that operations and injuries in the soles of the feet, and as a sequal to castration and the extripation of the thyroid gland for bronchocele, are operations in which the greatest tendency to this disease resides.

Wiess, reported thirteen cases of tetanus occurring after removal of the thyroid gland. In fifty-three total extripations of the thyroid glands for goitre, made by Billroth, tetanus followed in twelve cases, while no cases occurred in 109 partial operations.

Gautier has collected seventy-four cases of tetanus, thirty-six following abortion, and thirty-eight following confinement; autopsies were made in fifteen cases, three presented on microscopical examinations of the brain and cord, no appreciable lesion. In one case a retained putrified placeuta was found in the uterus, in five suppurative metritis, in one ovarian cyst, in one hemorrhage into the lateral ventricles. Ten patients recovered, five after abortion, five after labor.

As to symptoms, diagnosis, and other details of the same, I consider it unnecessary to deliniate before such a body as this, as all present are conversant with the symptoms of tetanus. There is only one thing, however, I would enjoin, that is, a too hasty diagnosis may result in a little embarrassment, as the writer has been twice deceived upon the first and hasty diagnosis.

In one case it was in a five year old mare, used for road purposes; she had gone lame three or four days previously. The owner sent the stable boy to the drug store, to procure a pound of ground flaxseed, he having other shopping to do returned to the store and took what he supposed to be his package. On arriving at the stable he made the necessary preparations to putting the lame foot into a poultice. In so doing he offered the animal a handful of his flaxseed, which he says she seemed to eat with delight. The poultice was applied at eleven o'clock. I was called to see the animal, she at that time was standing in a box, tied in opposite directions, right fore-foot pointing in a neatly prepared poultice, head and tail extended, saliva and froth issuing from the lips which were closed, ears erect and stiff. Body rigid and in convulsions and in a slight perspiration.

Upon raising the head and slightly tapping the neck I noticed that the membrana nictitans did not move over the orbit, as is so constant in tetanus, yet in the absence of that symptom I felt justified in my diagnosis. The only history I could get of the case is that already stated. I ordered poultice removed and parts washed, as I wished to examine the foot. At this point in the proceedings I was able to find the cause of these tetanic convulsions.

The poultice was made of powdered nux vomica, instead of ground flaxseed, and the quantity the animal had eaten was about ℥ ii.

In citing this case it is for the purpose of showing the physiological effect of this drug on the nervous centres and upon certain muscular groups.

As in tetanus we get like symptoms produced by the physiological effect of the bacillus, what they are capable of throwing off, and certain other peculiar substances "resembling alkoloids"

which are produced during the process of putrefaction of the dead ones in the system.

Regarding the treatment of this disease, nearly every known element that acts as a nervous sedative has been used to a greater or less extent with varying degrees of results; experience, however, leads me to believe that drugs play a minor part in the successful issue of the disease, and that no one drug can be relied upon as a panacea. That the disease must be treated according to the various stages of the disease, at the time we first see it, is to my mind, a very essential feature. If seen in the early stages when there is but partial rigidity of the voluntary muscles, and trismus is not perceptible, and deglutition is but little if any impaired; a thorough purge at this stage is the sheet-anchor of success, followed with Soda Hyposulphite and Carbolic acid, enjoining at the same time perfect quietude. If the patient is not seen until all the symptoms are well established, the jaws more or less firmly set and occasionally convulsions, to offer a purge at that stage must be guarded discriminately. However, if it can be administered without exciting the animal, it is beneficial. I have had the best success under these circumstances by administering subcutaneously Sulphate of Eserine and dilute Hydrocyanic Acid, by allowing the animal to drink alternately the Bromide of Potash and Hyposulphite of Soda.

If the disease is the result of an injury, thoroughly cleanse the wound and treat it antiseptically.

